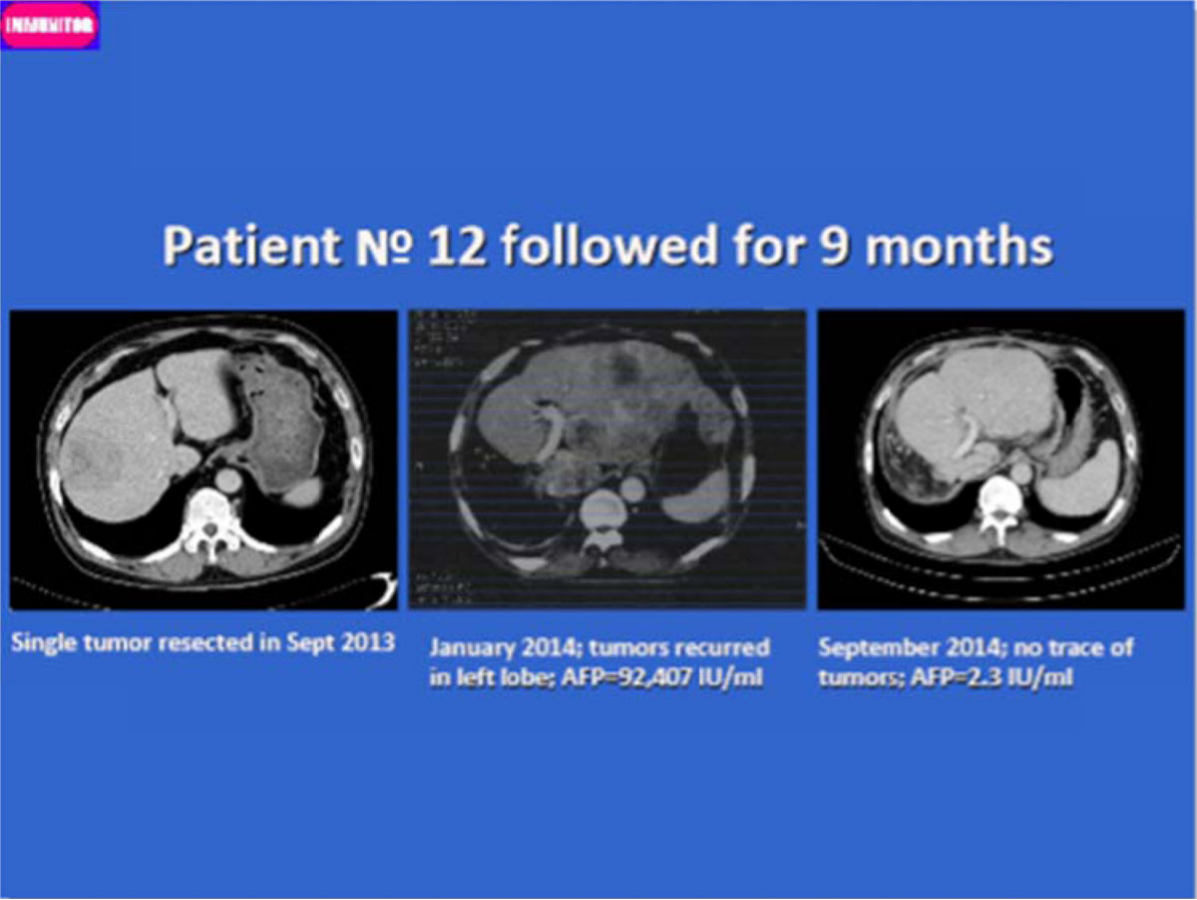# Immunotherapy of liver cancer with hepcortespenlisimut-L: open-label Phase II clinical study in patients with advanced HCC

**DOI:** 10.1186/2051-1426-3-S2-P200

**Published:** 2015-11-04

**Authors:** Marina G Tarakanovskaya, Jigjidsuren Chinburen, Genden Purevsuren, Chogsom Munkhzaya, Purev Batchuluun, Purev Bat-Ireedui, Dorjiin Dandii, Dandii Oyungerel, Galyna A Kutsyna, Allen I Bain, Vichai Jirathitikal, Aldar S Bourinbaiar

**Affiliations:** 1 Immunitor LLC, Ulaanbaatar, Mongolia; 2 National Cancer Center, Ulaanbaatar, Mongolia; 3 Monserum LLC, Ulaanbaatar, Mongolia; 4 National Center for Public Health, Ulaanbaatar, Mongolia; 5 Immunitor Inc, Vancouver, BC, Canada, V6K 2G8

## Introduction

Increasing number of studies is now devoted to immunotherapy of cancer. We evaluated the clinical benefit of hepcortespenlisimut‐L (V5) – an oral therapeutic vaccine designated by the US FDA as an orphan drug for treatment of hepatocellular carcinoma (HCC).

## Patients

Open-label Phase II trial (NCT02256514) is currently underway, which by now has enrolled 75 patients with advanced HCC, consisting of 29 (38.7%) females and 46 (61.3%) males with median age 60 years (Mean 61.6±8.1). Out of these 32 (42.7%) had hepatitis B and 43 (57.3%) hepatitis C infections, including 8 (10.7%) with dual infection, 4 (5.3%) negative for both viruses and 5 (6.7%) without established viral diagnosis.

## Results

After median 2 months of daily dose of V5 pill, 50 out 75 patients had experienced decline in serum levels of tumor marker, alphafetoprotein, (66.7%; P=0.008 by Wilcoxon Signed Rank test). Baseline median AFP levels were 245.2 IU/ml (Mean 4,233; Range 7.2-92,407; 95% CI 1,186-7,280) and post-treatment values were 102.3 IU/ml (Mean 2,539; Range 0.9-54,478; 95% CI 503-4,575). The decrease in AFP was correlated either with tumor clearance or regression on CT scans. The median overall survival time could not be established since 70 out 75 (93.3%) are still alive after median follow-up of 10 months (Mean 13; Range 5-57; 95% CI 10.7-15.2). The first patient in this study received immunotherapy 57 months ago and is doing well without any trace of lesions. None of the patients experienced any adverse effects, contrary their liver function tests had improved.

## Conclusion

The results indicate that hepcortespenlisimut-L is safe, effective and fast-acting immunomodulatory intervention for HCC. The Phase III randomized, double-blind, placebo-controlled trial is now initiated to confirm these promising findings (NCT02232490).

## Trial registration

ClinicalTrials.gov identifier NCT02232490.

**Figure 1:**